# *Gerhardtia tomentosa* and *Ossicaulis borealis* (Agaricales, Lyophyllaceae)—Two new species from northeast China

**DOI:** 10.3389/fmicb.2023.1118853

**Published:** 2023-04-05

**Authors:** Yue Qi, Fei Xu, Zhao-Qian Yang, Jian-Xuan Hou, Ai-Guo Xu, Hong-Bo Guo, Xiao-Dan Yu, Rui-Heng Yang

**Affiliations:** ^1^College of Biological Science and Technology, Shenyang Agricultural University, Shenyang, China; ^2^Alpine Fungarium, Tibet Plateau Institute of Biology, Lhasa, China; ^3^College of Life Engineering, Shenyang Institute of Technology, Fushun, China; ^4^Institute of Edible Fungi, Shanghai Academy of Agricultural Sciences, Shanghai, China

**Keywords:** Lyophyllaceae, *Gerhardtia*, *Ossicaulis*, phylogeny, morphology, taxonomy

## Abstract

**Background:**

*Gerhardtia and Ossicaulis* are two genera within the family Lyophyllaceae, which show an apparently poor species diversity worldwide. During the field investigation on wild macrofungi, six interesting collections within *Gerhardtia and Ossicaulis* genera are discovered in the northeastern China.

**Methods:**

To identify whether these collections of Gerhardtia and Ossicaulis are novel species, we performed phylogenetic analyzes using the following DNA regions: the internal transcribed spacer (ITS) region and the large subunit nuclear ribosomal RNA (nrLSU) region. Moreover, a traditional morphological method also be conducted based on both the macro-morphological and micro-morphological features.

**Results:**

The results indicated that these collections tested formed two independent lineages in each genus with a high support. In addition, they can easily be separated from all other taxa of the two genera in morphology. Based on the combination of morphological and molecular data, *Gerhardtia tomentosa* and *Ossicaulis borealis*, are confirmed as two new species to science.

**Discussions:**

This study provided a theoretical basis is for the two lyophylloid genera and indicated that the biodiversity resources of northeastern China might be underestimated.

## 1. Introduction

Lyophyllaceae, a highly species-rich family of mushrooms, includes *Gerhardtia* Bon and *Ossicaulis* Redhead & Ginns, two genera poor in species. The genus *Gerhardtia* (Lyophyllaceae, Agaricales) is a fungal taxon with *G. borealis* (Fr.) Contu and Ortega (2002; p. 176) [syn. *G. incarnatobrunnea* (Ew. Gerhardt) Contu and Ortega (2002; p. 66)] as a type species (Bon, 1994; Contu and Ortega, 2002; Vizzini et al., 2015). It was initially considered as a subgenus under *Lyophyllum* P. Karsten (1881; p. 29) (Karsten, 1881; Gerhardt, 1982) and later was raised to the genus level by Bon (1994) who emphasized the presence of minutely verruculose basidiospores, siderophilous basidia, pileipellis organized as a cutis, trichoderm or hymeniderm, and clampless hyphae as the generic concepts of *Gerhardtia*. Due to the discovery of *G. pseudosaponacea* Cooper and Leonard with a smooth surface of basidiospores (Cooper, 2014), the morphological circumscriptions of *Gerhardtia* were redefined as having basidiospores that are smooth or slightly verrucose (Vizzini et al., 2015). Subsequently, the generic definitions were partly amended again with a particular emphasis on basidiospores that are irregular and undulate to nodulose rather than verrucose (Vizzini et al., 2017).

*Ossicaulis* (Lyophyllaceae, Agaricales) is a very small genus and currently contains only four species: *O. lachnopus* (Fr.) Contu, *O. lignatilis* (Pers.) Redhead and Ginns, *O. yunnanensis* L.P. Tang, N.K. Zeng and S.D. Yang, and *O. salomii* Siquier and Bellanger (Contu, 2007; Holec and Kolarík, 2013; Yang et al., 2018; Crous et al., 2019). The genus is distinguished by brown-rot fungus, adnexed, adnate, or at most subdecurrent lamellae, central to the eccentric stipe, regular trama, small and ellipsoid basidiospores, coralloid hyphae in the epicutis, presence or absence of cheilocystidia, and presence of clamp connections (Holec and Kolarík, 2013).

In this study, six mushroom collections from northeast China, two of *Gerhardtia* and four of *Ossicaulis*, were analyzed and described. Molecular phylogeny based on the combined ITS and nLSU datasets revealed that these collections occupied two independent lineages in each genus. In addition, there are distinctive morphological differences between the two species and all other taxa of the two genera. Detailed descriptions of the two lyophylloid new species are reported here.

## 2. Materials and methods

### 2.1. Sampling and morphology

The basidiomes were photographed in the field. Dried specimens were preserved in the Fungal Herbarium of Shenyang Agricultural University (SYAU-FUNGI), Liaoning, China. The voucher numbers of the specimens collected in this study are from SYAU-FUNGI-074 to SYAU-FUNGI-079 (Supplementary Table 1). Tissue blocks were removed from dried specimens for DNA analyses. Colors are coded based on the study by Kornerup and Wanscher (1963). Methods for morphological descriptions followed Pei et al. (2021).

### 2.2. DNA extractions, amplification, and sequencing

Genomic DNA was extracted from the dried specimens using the cetyltrimethylammonium bromide (CTAB) method (Doyle and Doyle, 1987). The universal primer pairs ITS1/ITS4 (White et al., 1990) and LROR/LR5 (Michot et al., 1984) were used for the amplification of the ITS and nrLSU regions, respectively. The PCR protocol and sequencing were conducted, as described by Pei et al. (2021). The newly generated sequences were submitted to GenBank (Supplementary Table 1).

### 2.3. Sequence alignment and phylogenetic analyses

**Figure 1:**
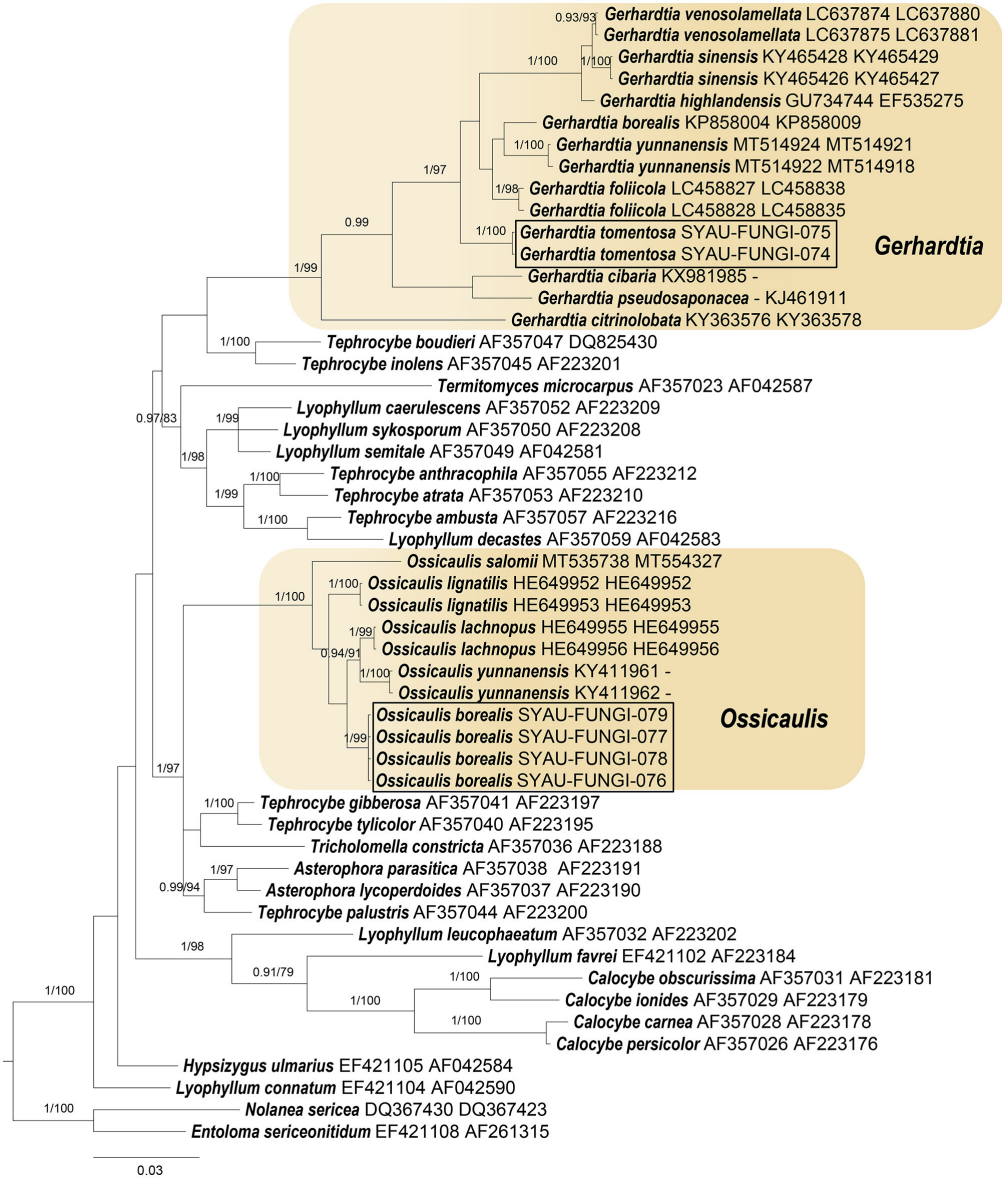
ML and BI analysis of Lyophyllaceae s.l. based on ITS and nrLSU sequences. PP ≥0.95 **(left)** and BS ≥60 **(right)** are indicated on branches. *Entoloma sericeonitidum* and *Nolanea sericea* are rooted as the out-groups. Newly generated sequences for *Gerhardtia tomentosa* and *Ossicaulis borealis* are highlighted in boxes. GenBank accession numbers are provided after the species name.

**Figure 2:**
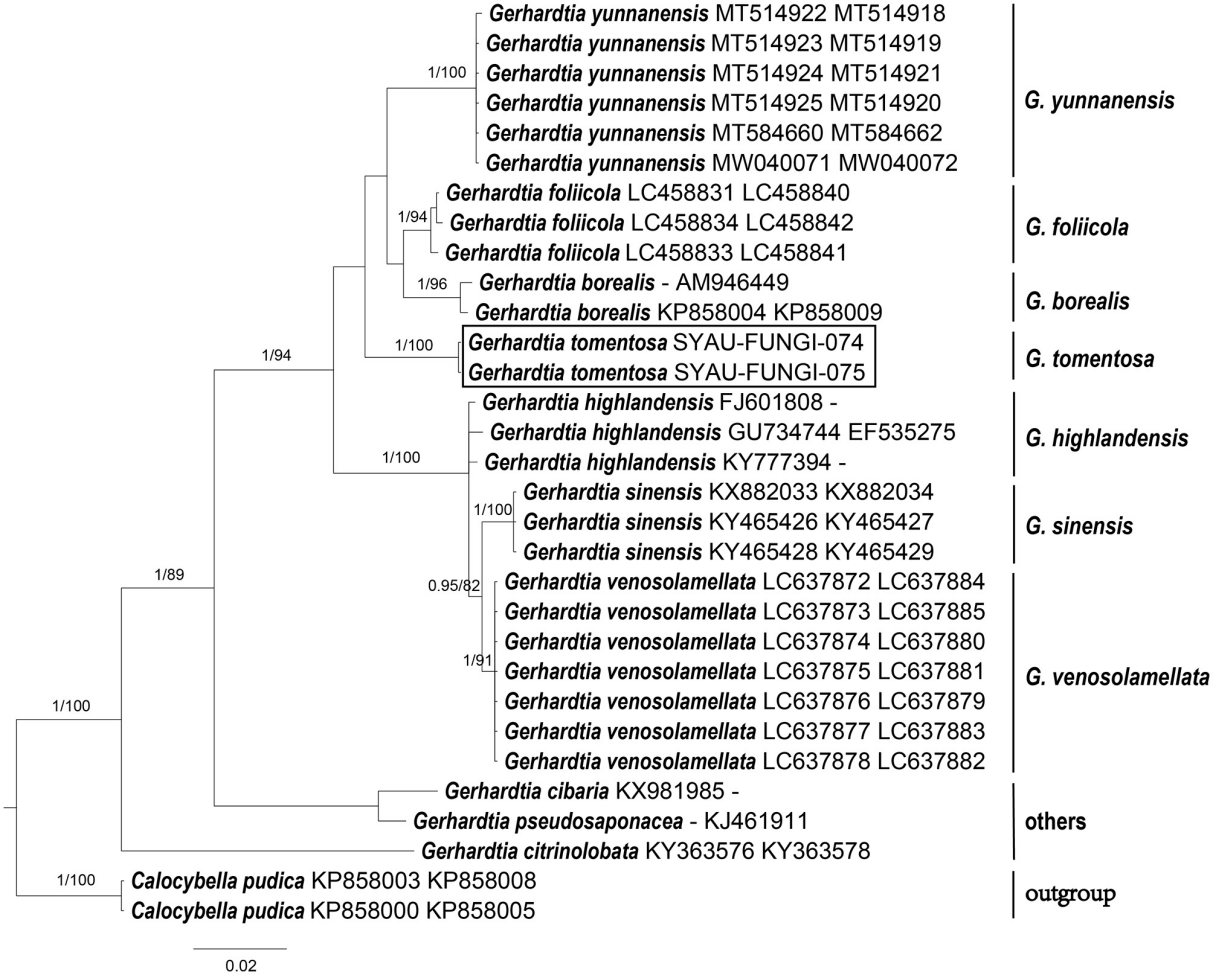
ML and BI analysis of *Gerhardtia* based on ITS and nrLSU sequences. PP ≥0.95 **(left)** and BS ≥60 **(right)** are indicated on branches. *Calocybella pudica* is rooted as the out-group. Newly generated sequences for *Gerhardtia tomentosa* are highlighted in boxes. GenBank accession numbers are provided after the species name.

**Figure 3:**
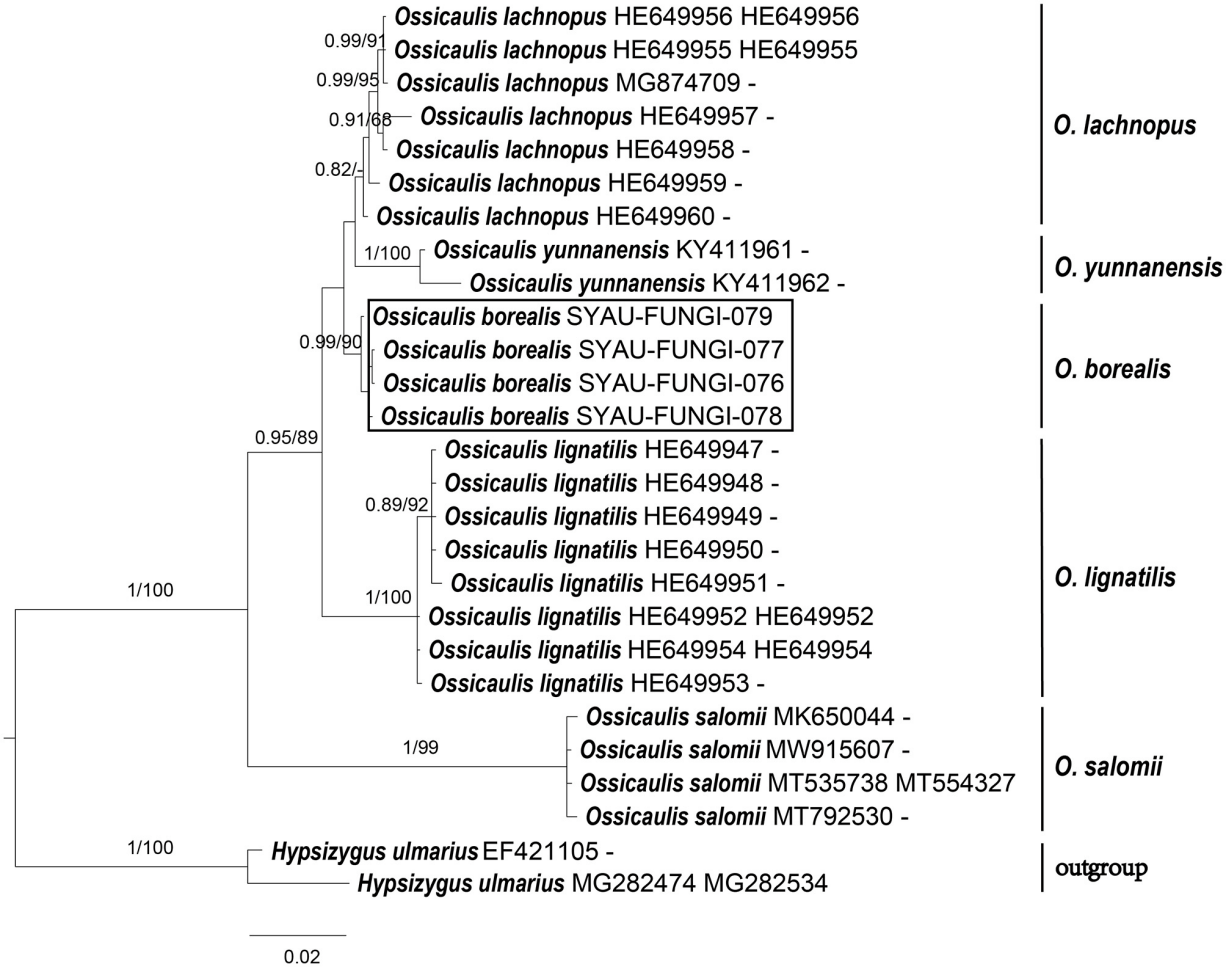
ML and BI analysis of *Ossicaulis* based on ITS and nrLSU sequences. PP ≥0.95 **(left)** and BS ≥60 **(right)** are indicated on branches. *Hypsizygus ulmarius* is rooted as the out-group. Newly generated sequences for *Ossicaulis borealis* are highlighted in boxes. GenBank accession numbers are provided after the species name.

The sequences obtained in this study were checked and edited using Bioedit v7.0.9 (Hall, 1999) and aligned with those available in GenBank using Blastn. According to the Blastn results and outcomes of recent phylogenetic studies on Lyophyllaceae (Mešic and Tkalcec, 2009; Bellanger et al., 2015; Li et al., 2017; Matheny et al., 2017; Endo et al., 2019, 2021; Mu et al., 2021), high-quality sequences were downloaded from GenBank databases. Alignments were, then, generated for each single ITS and nrLSU dataset using MAFFT v7.313 (Katoh and Standley, 2013). Three combined alignments comprising ITS and nrLSU regions were generated after the concatenation of single ITS and nrLSU datasets. *Calocybella pudica* for the genus *Gerhardtia, Hypsizygus ulmarius* for the genus *Ossicaulis*, as well as *Entoloma sericeonitidum* and *Nolanea sericea* for the family Lyophyllaceae were selected as outgroup taxa. The three combined alignments were then imported into PAUP 4.0b4a (Swofford, 2003) for partition homogeneity tests (Farris et al., 1995), and the results indicated that ITS and nrLSU regions can be combined for phylogenetic studies (*P*-value = 0.71 for Lyophyllaceae; *P*-value = 0.98 for *Gerhardtia*; *P*-value = 0.77 for *Ossicaulis*). Bayesian inference (BI) and maximum likelihood (ML) were employed to infer the phylogenetic position of the two species. The RAxML-8.2.10-WIN was used to infer the ML tree, with the GTRGAMMA as a default model (Stamatakis, 2014). The MrBayes v.3.2.6 was used to infer the BI tree, with the GTR+I+G as a best-fitting evolution model (Ronquist et al., 2012). The three combined alignments were run for 2,000,000 generations with four chains, and trees were sampled every 1,000 generations. The best trees were viewed in FIGTREE v1.4.4 (Rambaut, 2018) and were compiled in Adobe Illustrator CC (Figures 1–3).

## 3. Results

### 3.1. Molecular phylogenetic results

For the construction of phylogenetic relationships, three combined datasets for Lyophyllaceae, *Gerhardtia*, and *Ossicaulis* were generated as described earlier. Dataset 1 for Lyophyllaceae comprised 52 sequences (six determined in this study), and it was 1,400 bp long; dataset 2 for *Gerhardtia* includes 31 sequences (two determined in this study), and it was 1,227 bp long; and dataset 3 for *Ossicaulis* includes 27 sequences (four determined in this study), and it was 1,313 bp long. The almost identical tree topologies were recovered using both the BI and ML approaches in this study, and only the BI trees were displayed with PP and BS values for nodes (Figures 1–3). The phylogenetic analysis of dataset 1 showed the monophyly of Lyophyllaceae with high support (PP = 1, BS = 100) and also suggested that *Gerhardtia* and *Ossicaulis* should belong to monophyletic groups (PP = 1, BS = 99 for *Gerhardtia*; PP = 1, BS = 100 for *Ossicaulis*). According to the phylogram of Lyophyllaceae (Figure 1), two new species occupied an independent position in *Gerhardtia* and *Ossicaulis*, respectively, with strong statistical support (PP = 1, BS = 100 for *Gerhardtia tomentosa*; PP = 1, BS = 99 for *Ossicaulis borealis*). The phylograms of *Gerhardtia* and *Ossicaulis* resulting from the analysis of dataset 2 and dataset 3, respectively, also confirmed the independent positions of the two new species with high support (Figures 2, 3).

### 3.2. Taxonomy

**Figure 4:**
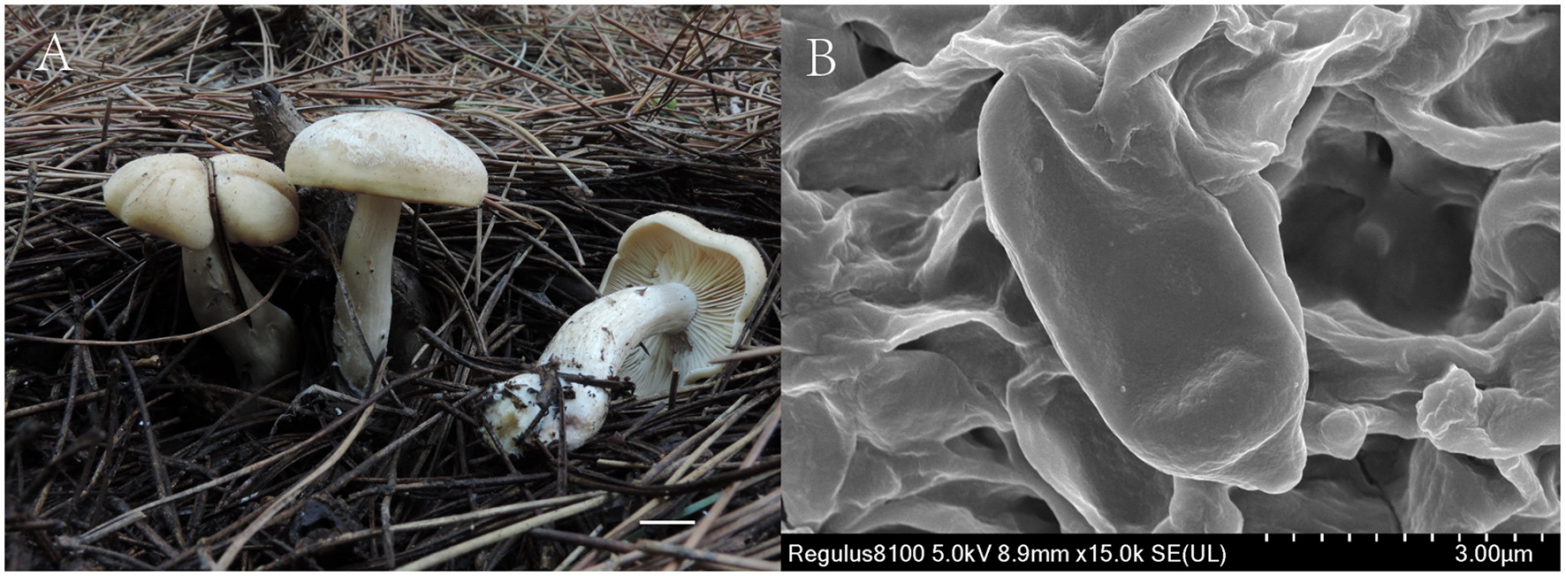
*Gerhardtia tomentosa* (Holotype, SYAU-FUNGI-074). **(A)** macroscopic habitat **(B)** surface of basidiospores. Scale bars: 1 cm **(A)**; 3 μm **(B)**.

**Figure 5:**
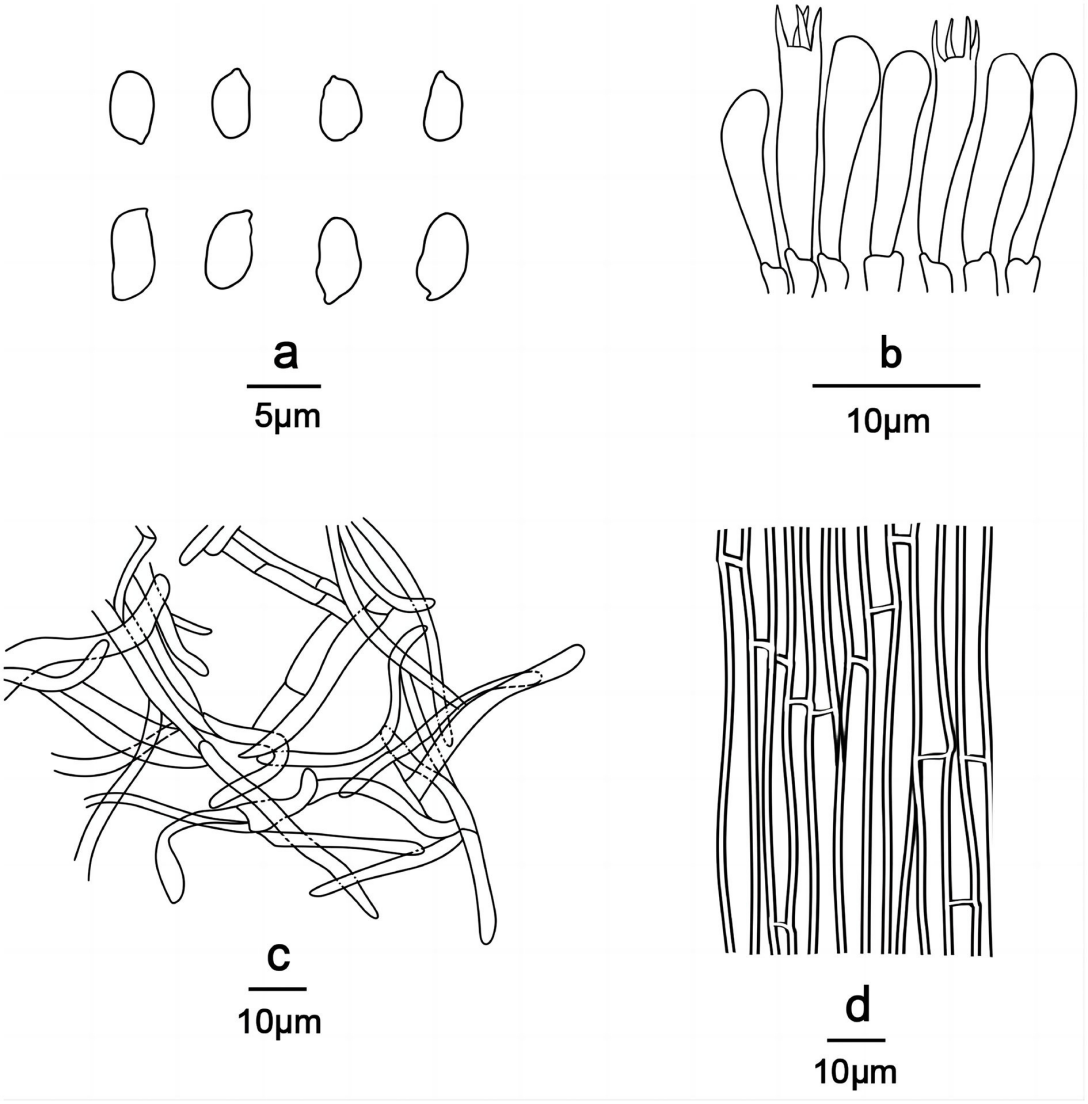
Line drawings of *Gerhardtia tomentosa* (Holotype, SYAU-FUNGI-074). **(a)** Basidiospores, **(b)** basidia and basidioles, **(c)** Pileipellis, and **(d)** hyphae from trama.

***Gerhardtia tomentosa*** X.D. Yu & H.B. Guo, sp. nov. Figures 4, 5.

MycoBank No. MB 846452.

**Etymology**: The epithet “*tomentosa*” refers to the minute, white tomentose pileus surface.

**Diagnosis**: Distinguished by its tricholomatoid basidiomata, cream to yellowish gray pileus with the tomentose surface, stipe with white basal tomentum, ellipsoid to cylindrical, smooth or minutely verrucose basidiospores, (5.0–) 5.5–6.5 (−7.2) × (1.8–) 2.0–3.0 (−3.2) μm, and absence of cystidia.

**Type:** CHINA. Liaoning Province: Shenyang City, Shenyang Institute of Applied Ecology, on the soil in meadows, 30 Sep. 2012, X.D. Yu (holotype: SYAU-FUNGI-074).

**Description**: Basidiomata medium-sized. Pileus 45–65 mm broad, slightly hemispherical to convex, then expanding to plano-convex when mature; margin regular to wavy, not translucently striate; surface dry, dull, non-smooth, with minutely, whitish tomentous, especially when young and then with a cream (4A2 to 4A3) to yellowish gray (4B2 to 4B3) coating, cracked with time, exposing white background. Lamellae 4–7 mm broad, adnate to adnexed, moderately crowded, with several levels of lamellae, concolorous with pileus, with entire to undulate, concolorous edge. Stipe 30–65 mm × 10–15 mm diam., central, solid, subcylindrical, or tapering toward the base, sometimes thickened in the middle, whitish to light brown (5D4), finely tomentose at the base. Context white. Odor and taste not distinctive. Spore deposit creamy.

Basidiospores (85/6/5) (5.0–) 5.5–6.5 (−7.2) × (1.8–) 2.0–3.0 (−3.2) μm, Q = 1.55–2.13, Qm = 1.98 ± 0.10, ellipsoid to cylindrical, thin-walled, hyaline, cyanophilous, inamyloid, apiculus small, approximately 0.6 μm long, basidiospores wall smooth and then slightly undulate under the light microscope, sometimes with very minute verrucae under SEM. Basidia 25.0–35.0 × 5.0–7.0 μm, narrowly clavate or cylindrical, thin-walled, with cyanophilous and siderophilous granulations, four-spored, with sterigmata up to 2.0–3.0 μm long. Cheilocystidia and pleurocystidia absent. Hymenophoral trama composed of regular, densely parallel hyphae, and hyphae 4.0–9.0 μm wide. Pileipellis, a cutis composed of interwoven hyphae, hyphae 6.0–7.0 μm wide. Stipitipellis similar to the pileipellis, hyphae 6.0–7.0 μm wide. Clamp connections absent.

**Habitat and distribution**: Scattered on the soil, on the grass, and in the woods. Known only from Liaoning province in northeastern China.

**Additional material studied**: CHINA. Liaoning Province: Shenyang City, Dongling Park, on the soil in meadows, 21 Jul. 2019, X.D. Yu (SYAU-FUNGI-075).

**Notes**: *Gerhardtia tomentosa* can be separated from most *Gerhardtia* species by the pileus color—*Gerhardtia borealis* (Bon, 1994), *G. cibaria* (Matheny et al., 2017), *G. foliicola* (Endo et al., 2019), *G. leucopaxilloides* (Bigelow and Smith, 1969), and *G. yunnanensis* (Mu et al., 2021) have brownish pileus; *G. venosolamellata* (Endo et al., 2021) and *G. highlandensis* (Bigelow and Smith, 1969) have white to pale pink pileus; *G. citrinolobata* (Vizzini et al., 2017) is characterized by a lemon yellow pileus; *G. suburens* (Clémençon, 1968) has a grayish pileus, and *G. marasmioides* (Singer and Digilio, 1952) possesses an ochraceous pileus, which differs from *G. tomentosa*, characterized by a cream to yellowish gray pileus. *Gerhardtia piperata* (Mešic and Tkalcec, 2009), *G. pseudosaponacea* (Cooper, 2014; Vizzini et al., 2015), and *G. sinensis* (Li et al., 2017) have a great resemblance to *G. tomentosa* because all of them have a pale yellow pileus and tricholomatoid basidiomata; however, *G. piperata* can be delimited by its much larger basidiomata up to 120 mm broad, longer basidiospores up to 7.8 μm, and more undulate spore surface (Mešic and Tkalcec, 2009); *G. pseudosaponacea* can distinguish from *G. tomentosa* by its possession of glabrous stipe base, closer lamellae, cheilocystidia and basidiospores with a smooth surface (outline) under the light microscope (Cooper, 2014; Vizzini et al., 2015); *G. sinensis* differs from *G. tomentosa* by its distant and intervenose lamellae as well as basidiospores with a smooth surface (outline) under the light microscope (Li et al., 2017).

**Figure 6:**
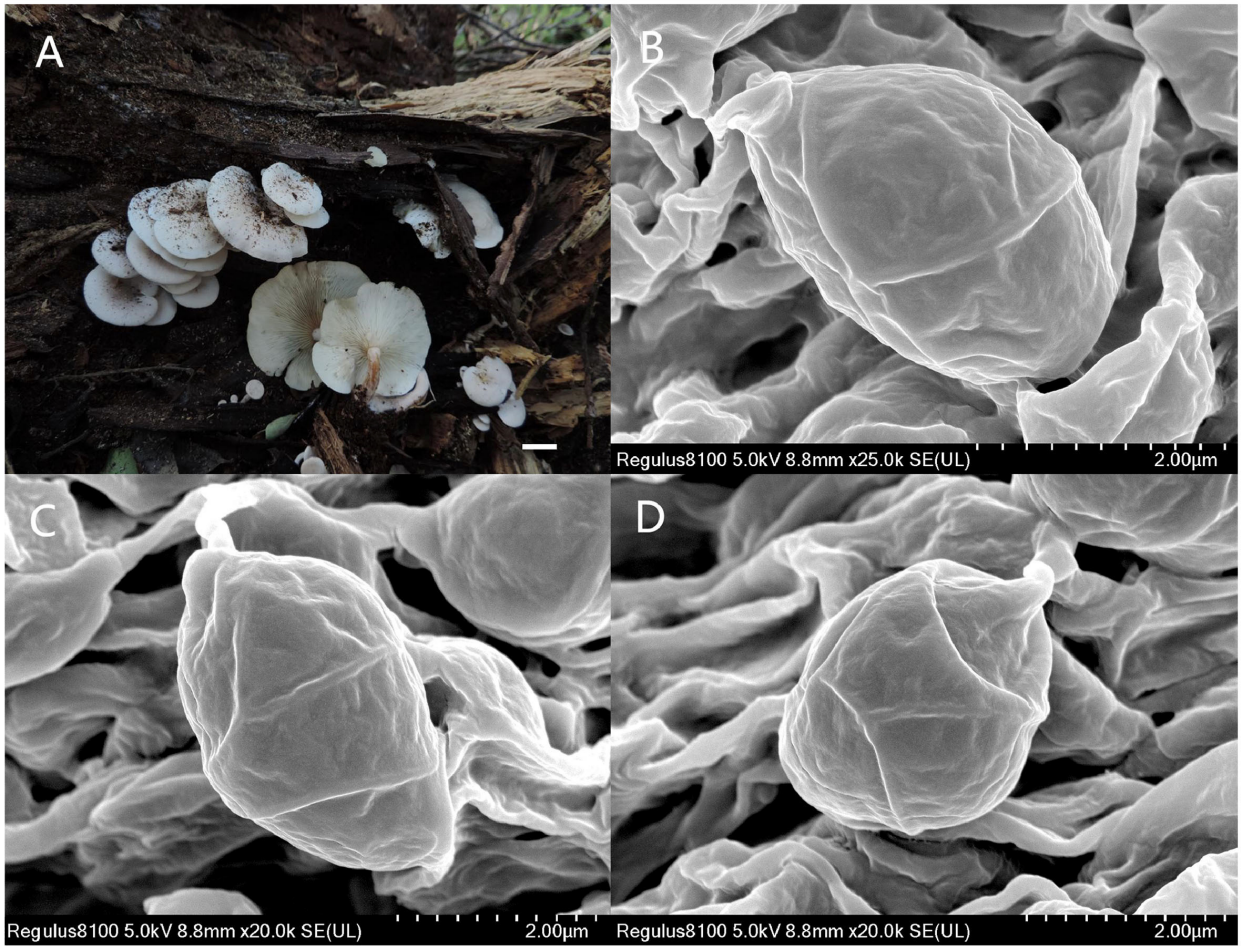
*Ossicaulis borealis* (Holotype, SYAU-FUNGI-076). **(A)** Macroscopic habitat and **(B,C,D)** surface of basidiospores. Scale bars: 1 cm **(A)**; 2 μm **(B)**.

**Figure 7:**
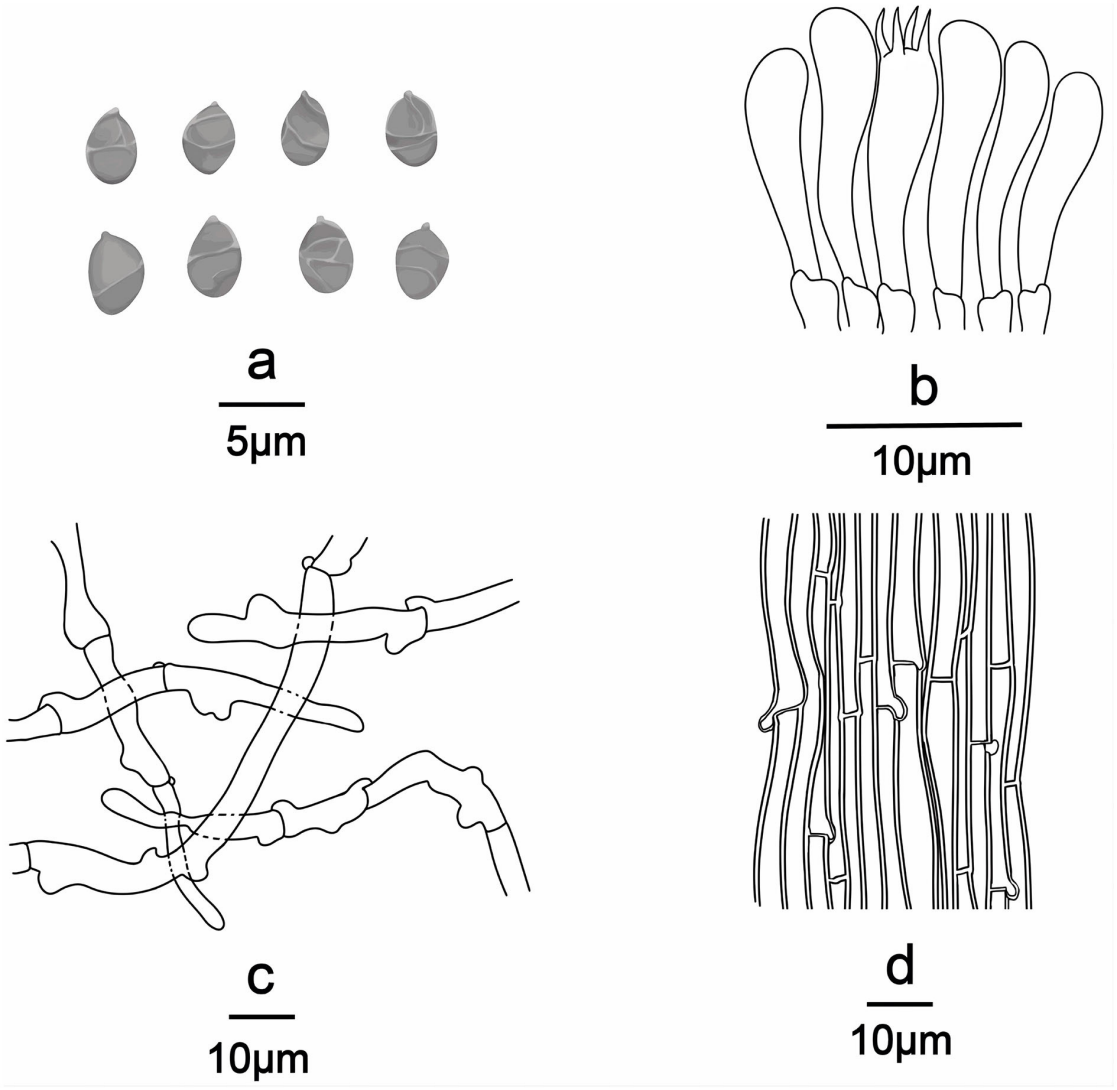
Line drawings of *Ossicaulis borealis* (Holotype, SYAU-FUNGI-076). **(a)** Basidiospores, **(b)** basidia and basidioles, **(c)** Pileipellis, and **(d)** hyphae from trama.

***Ossicaulis borealis*** X.D. Yu & H.B. Guo, sp. nov. Figures 6, 7.

MycoBank no. MB **846453**.

**Etymology:** The epithet “*borealis*” refers to the northern part of China, the holotype locality.

**Diagnosis:** Distinguished by its medium to large basidiomata, white to grayish orange or brownish orange pileus, central to the eccentric and often curved stipe, ellipsoid basidiospores ornamented with irregularly reticulate ridges, (2.8–) 3.0–4.0 × (−1.8) 2.0–2.4 (−2.8) μm, and absence of cystidia.

**Type:** CHINA. Jilin Province: Yanbian autonomous prefecture, Baihe nature reserve, growing on the dying or decaying woods of deciduous trees in a broad-leaved forest, 4 Sep. 2019, X.D. Yu & H.B. Guo (SYAU-FUNGI-076).

**Description:** Basidiomata medium to large. Pileus 20–100 mm, hemispherical to convex at first, becoming applanate to depressed at the center when mature, sometimes even clitocyboid; margin entire when young, expanding to flexuous when mature, sometimes slightly lobed; surface white to grayish orange (5B3) to brownish orange (5C3 to 5C4). Lamellae thin, narrow, very crowded, adnate, adnexed, or subdecurrent, with several levels of lamellae; margin entire or flexuous, whitish when young, then becoming pale grayish orange (5B3) when mature. Stipe 20–80 mm × 3–10 mm diam., central to eccentric, solid to hollow, subcylindrical, slightly broadened toward the base, often curved; surface white to grayish orange (5B4 to 6B4) to brownish orange (6C4), white mycelium present at the base. Context thin, white. Odor and taste not distinctive. Spore deposit white.

Basidiospores (96/8/5) (2.8–) 3.0–4.0 × (−1.8) 2.0–2.4 (−2.8) μm, Q = 1.32–1.65, Qm = 1.45 ± 0.10, ellipsoid, thin-walled, hyaline, cyanophilous, inamyloid, apiculus small, approximately 0.3 μm long, smooth under the light microscope, with irregular ridges under SEM. Basidia 20.0–25.0 × 4.0–5.0 μm, narrowly clavate or cylindrical, thin-walled, with cyanophilous and siderophilous granulations, four-spored, with sterigmata up to 2.0–3.0 μm long. Cheilocystidia and pleurocystidia absent. Hymenophoral trama composed of regular, densely parallel hyphae, hyphae 4.0–10.0 μm wide. Pileipellis, a trichoderm composed of interwoven hyphae, hyphae 6.0–8.0 μm wide, sometimes with obtuse tubercles to short outgrowths. Stipitipellis similar to the pileipellis, hyphae 6.0–9.0 μm wide. Clamp connections present in all tissue.

**Habitat and distribution:** Cespitose, on the dying or decaying woods of deciduous trees, in a mesotemperate forest. Known only from Jilin province, Heilongjiang province, and Inner Mongolia in northern China.

**Additional material studied:** CHINA. Jilin Province: Yanbian Korean autonomous prefecture, Baihe Nature Reserve, growing on the dying or decaying woods of deciduous trees, 19 Aug. 2019, X.D. Yu & H.B. Guo (SYAU-FUNGI-077); CHINA. Inner Mongolia: Arxan National Forest Park, growing on the dying or decaying woods of deciduous trees, 18 Aug. 2017, X.D. Yu (SYAU-FUNGI-078); CHINA. Heilongjiang Province: Yichun City, Wuying National Forest Park, growing on the dying or decaying woods of deciduous trees, 28 Aug. 2015, H.B. Guo (SYAU-FUNGI-079).

**Notes:** *Ossicaulis borealis* resembles *O. lachnopus* and *O. lignatilis* from Europe as well as *O. yunnanensis* from China in macro-morphological features. However, *O. borealis* can be delimited from the three taxa in the following ways. Considering the size of basidiospores, *O. lignatilis* is characterized by relatively larger basidiospores (4.0–5.6 × 2.4–3.6 μm), and *O. yunnanensis* has slightly smaller basidiospores (2.6–3.0 × 1.8–2.0 μm) than *O. borealis* (3.0–4.0 × 2.0–2.4 μm) (Holec and Kolarík, 2013; Yang et al., 2018). In terms of habitat, *O. yunnanensis* grows on the living tree trunk of *Rhododendron*, whereas *O. borealis* grows on the dying or decaying tree trunks such as *O. lachnopus* and *O. lignatilis* (Holec and Kolarík, 2013; Yang et al., 2018). Furthermore, the absence of all types of cystidia can also make *O. borealis* distinguishable from these three *Ossicaulis* species (Crous et al., 2019). *Ossicaulis salomii*, originally discovered in Spain, is very easily distinguishable by its much smaller basidiocarps approximately only 11 mm broad, pileus with white rimulose coating, larger basidiospores (4.0–5.0 × 3.0–4.0 μm), unique habitat next to the sea, and so on (Crous et al., 2019).

## 4. Discussion

Basidiomata morphology and molecular data suggest that *Gerhardtia tomentosa* and *Ossicaulis borealis* are two new species to science. Morphological differences between the two new species and other *Gerhardtia* and *Ossicaulis* species are described in detail. Phylogenetic analyses based on ITS and nrLSU regions indicate that *Gerhardtia* and *Ossicaulis* belong to monophyletic groups (Figures 1–3), which are consistent with the result of the comprehensive phylogenetic overview of Lyophyllaceae (Bellanger et al., 2015). In addition, collections of the two species occupy two independent lineages in each genus (Figures 1–3). *Gerhardtia tomentosa*, far from other species of *Gerhardtia*, is relatively close to the three taxa—*Gerhardtia foliicola, G. yunnanensis*, and *G. borealis*. However, *Gerhardtia tomentosa* could differ from them by a whitish pileus. *G. foliicola* (Endo et al., 2019) has brown to dark brown pileus; *G. yunnanensis* (Mu et al., 2021) has yellowish to reddish brown pileus; and *G. borealis* (Bon, 1994) has reddish brown pileus. Furthermore, *G. foliicola* also differs by its slightly smaller pileus approximately 25–40 mm broad, very crowded lamellae, slightly shorter basidiospores (3.5–5.5 × 2.0–3.0 μm), and presence of cystidia covered with granules on living cultured mycelium (Endo et al., 2019); *G. yunnanensis* can be distinguished by its slightly larger pileus up to 160 mm broad, translucent-striate pileus margin, and presence of narrowly clavate to irregular or curved clavate cystidia (Mu et al., 2021); *G. borealis* differs by its much larger basidiospores (6.0–8.5 × 4.0–5.0 μm) (Vizzini et al., 2015). *Ossicaulis borealis* forms a separate clade sister to that containing *O. lachnopus* and *O. yunnanensis* with very low statistical support.

Until now, 20 species of *Gerhardtia* and 4 species of *Ossicaulis* have been validly published all over the world, according to Index Fungorum (2022). Among these species, only three taxa have been originally reported from China, including two species of *G. sinensis* and *G. yunnanensis* and one species of *O. yunnanensis*. The discovery of *G. tomentosa* and *O. borealis* extends the species diversity of the two poor lyophylloid genera, which also indicates that the biodiversity resources of northeastern China may be underestimated.

The genus *Gerhardtia* is characterized by a small to large, collybioid, tricholomatoid, or marasmioid basidiomata with pileus ranging from white to pale pink, grayish, lemon yellow, pale yellow, and yellowish to reddish brown; pileipellis organized as either a cutis, trichoderm, or hymeniderm; basidiospores with the smooth or slightly undulate surface (outline) under the light microscope, and presence or absence of cystidia (Mešic and Tkalcec, 2009; Li et al., 2017; Matheny et al., 2017; Endo et al., 2019, 2021; Mu et al., 2021). The morphological heterogeneity suggests that these characteristics may be only significant at the infrageneric level rather than the generic level; the genus lacks uniform morphological features to be used as the generic definition. With the exception of *O. salomii*, the other four *Ossicaulis* species share many common characteristics, making it difficult to distinguish these taxa with morphology methods (Redhead and Ginns, 1985; Holec and Kolarík, 2013; Yang et al., 2018; Crous et al., 2019). In addition, molecular data available on GenBank databases are insufficient, especially molecular markers of protein coding genes, such as *rpb2* and *tef1*α, which restrict the multi-gene phylogenetic studies of *Ossicaulis*. Therefore, further field investigations and molecular studies on these two lyophylloid genera are still needed.

## Data availability statement

The datasets presented in this study can be found in online repositories. The names of the repository/repositories and accession number(s) can be found in the article/Supplementary material.

## Author contributions

X-DY and R-HY: conceptualization, validation, and funding acquisition. YQ and J-XH: methodology and software. A-GX: formal analysis. X-DY and H-BG: investigation. FX and Z-QY: resources. YQ: data curation and writing—original draft preparation. YQ and X-DY: writing—review and editing. H-BG and Z-QY: visualization. X-DY: supervision. R-HY: project administration. All authors have read and agreed to the published version of the manuscript.
