# Supplementary material for: Gerhardtia tomentosa and Ossicaulis borealis (Agaricales, Lyophyllaceae)—Two new species from northeast China
Source: Front Microbiol. 2023 Apr 5;14:1118853. doi: 10.3389/fmicb.2023.1118853 (PMC10115223; doi:10.3389/fmicb.2023.1118853)
Supplement: Supplementary file 1 [file Table_1.DOCX]

Supplementary Material

Description of two new species within two lyophylloid genera from China based on morphological and phylogenetic studies

Yue Qi^1^, Ai-Guo Xu^3^, Hong-Bo Guo^4^, Fei Xu^1^, Zhao-Qian Yang^1^, Jian-Xuan Hou^1^, Xiao-Dan Yu^1*^, Rui-Heng Yang^2*^

*** Correspondence:**

Xiao-Dan Yu, Rui-Heng Yang

[yuxd126@126.com;](mailto:yuxd126@126.com;) yangruiheng@126.com

**Supplementary Table 1.** Sequences used in phylogenetic analyses.

| **Species** | **Voucher** | **Genbank Accession Numbers** | |
| --- | --- | --- | --- |
|  |  | **ITS** | **nrLSU** |
| *Asterophora parasitica* | CBS683.82 | AF357038 | AF223191 |
| *Asterophora lycoperdoides* | CBS170.86 | AF357037 | AF223190 |
| *Calocybe carnea* | CBS552.50 | AF357028 | AF223178 |
| *Calocybe persicolor* | HC80/99 | AF357026 | AF223176 |
| *Calocybe obscurissima* | HC79/181 | AF357031 | AF223181 |
| *Entoloma sericeonitidum* | TB7144 | EF421108 | AF261315 |
| *Calocybe ionides* | HC77/133 | AF357029 | AF223179 |
| *Calocybella pudica* | AMB15994 | KP858000 | KP858005 |
| *Calocybella pudica* | AMB15997 | KP858003 | KP858008 |
| *Gerhardtia venosolamellata* | TUMH63956 | LC637874 | LC637880 |
| *Gerhardtia venosolamellata* | TUMH64253 | LC637875 | LC637881 |
| *Gerhardtia venosolamellata* | TUMH64252 | LC637876 | LC637879 |
| *Gerhardtia venosolamellata* | TUMH64255 | LC637877 | LC637883 |
| *Gerhardtia venosolamellata* | TUMH 64254 | LC637878 | LC637882 |
| *Gerhardtia citrinolobata* | JBSD126508 | KY363576 | KY363578 |
| *Gerhardtia borealis* | AMB15993 | KP858004 | KP858009 |
| *Gerhardtia borealis* | U.Soderholm 1593(H) | - | AM946449 |
| *Gerhardtia yunnanensis* | MHKMU-HT050 | MT514922 | MT514918 |
| *Gerhardtia yunnanensis* | MHKMU-Tang2542 | MT514923 | MT514919 |
| *Gerhardtia yunnanensis* | MHKMU-Tang2686 | MT514924 | MT514921 |
| *Gerhardtia yunnanensis* | MHKMU-Pu026 | MT514925 | MT514920 |
| *Gerhardtia yunnanensis* | MHKMU-Yang412 | MT584660 | MT584662 |
| *Gerhardtia yunnanensis* | MHKMU-Huang609 | MW040071 | MW040072 |
| *Gerhardtia foiicola* | TUMH63314 | LC458831 | LC458840 |
| *Gerhardtia foiicola* | TUMH63262 | LC458833 | LC458841 |
| *Gerhardtia foiicola* | TUMH63816 | LC458834 | LC458842 |
| *Gerhardtia highlandensis* | TENN-F-016020 | FJ601808 | - |
| *Gerhardtia highlandensis* | PBM2806 | GU734744 | EF535275 |
| *Gerhardtia highlandensis* | TENN-F-070609 | KY777394 | - |
| *Gerhardtia cibaria* | As. s.n. | KX981985 | - |
| *Gerhardtia sinensis* | GDGM46394 | KX882033 | KX882034 |
| *Gerhardtia sinensis* | GDGM42158 | KY465426 | KY465427 |
| *Gerhardtia sinensis* | GDGM45221 | KY465428 | KY465429 |
| ***Gerhardtia tomentosa*** | **SYAU-FUNGI-074** | **OP782037** | **OP782051** |
| ***Gerhardtia tomentosa*** | **SYAU-FUNGI-075** | **OP782038** | **OP782052** |
| *Gerhardtia pseudosaponacea* | PDD96650 | - | KJ461911 |
| *Hypsizygus ulmarius* | DUKE-JM/HW | EF421105 | - |
| *Hypsizygus ulmarius* | P135 | MG282474 | MG282534 |
| *Lyophyllum caerulescens* | HC80/140 | AF357052 | AF223209 |
| *Lyophyllum decastes* | JM87/16(T1) | AF357059 | AF042583 |
| *Lyophyllum sykosporum* | IFO30978 | AF357050 | AF223208 |
| *Lyophyllum semitale* | HC85/13 | AF357049 | AF042581 |
| *Lyophyllum favrei* | IEBSGHC 96cp4 | EF421102 | AF223184 |
| *Lyophyllum leucophaeatum* | Hae 251.97 | AF357032 | AF223202 |
| *Lyophyllum connatum* | DUKE-JM90c | EF421104 | AF042590 |
| *Nolanea sericea* | VHAs03/02 | DQ367430 | DQ367423 |
| *Ossicaulis salomii* | JLS3421 | MK650044 | - |
| *Ossicaulis lachnopus* | PRM:899407 | HE649957 | - |
| *Ossicaulis lachnopus* | 3855 | MG874709 | - |
| *Ossicaulis salomii* | EGDA-OS2 | MW915607 | - |
| *Ossicaulis salomii* | [Ghobad-Nejhad 4324](https://www.ncbi.nlm.nih.gov/nuccore/MT554327.1) | MT535738 | MT554327 |
| *Ossicaulis salomii* | EGDA-OS3 | MT792530 | - |
| *Ossicaulis lachnopus* | PRM537802 | HE649960 | - |
| *Ossicaulis lachnopus* | PRM824708 | HE649959 | - |
| *Ossicaulis lachnopus* | PRM899418 | HE649958 | - |
| *Ossicaulis lachnopus* | PRM899221 | HE649956 | HE649956 |
| *Ossicaulis lachnopus* | PRM899181 | HE649955 | HE649955 |
| *Ossicaulis lignatilis* | PRM515902 | HE649947 | - |
| *Ossicaulis lignatilis* | PRM715628 | HE649948 | - |
| *Ossicaulis lignatilis* | PRM829164 | HE649949 | - |
| *Ossicaulis lignatilis* | PRM899405 | HE649950 | - |
| *Ossicaulis lignatilis* | PRM829198 | HE649951 | - |
| *Ossicaulis lignatilis* | PRM897367 | HE649952 | HE649952 |
| *Ossicaulis lignatilis* | PRM889513 | HE649954 | HE649954 |
| *Ossicaulis lignatilis* | PRM889177 | HE649953 | - |
| ***Ossicaulis borealis*** | **SYAU-FUNGI-076** | **OP782047** | **OP782284** |
| ***Ossicaulis borealis*** | **SYAU-FUNGI-077** | **OP782048** | - |
| ***Ossicaulis borealis*** | **SYAU-FUNGI-078** | **OP782049** | - |
| ***Ossicaulis borealis*** | **SYAU-FUNGI-079** | **OP782050** | **OP782285** |
| *Ossicaulis yunnanensis* | IH26 | KY411961 | - |
| *Ossicaulis yunnanensis* | IJ152 | KY411962 | - |
| *Tephrocybe inolens* | CBS330.85 | AF357045 | AF223201 |
| *Termitomyces microcarpus* | PRU3900 | AF357023 | AF042587 |
| *Tephrocybe anthracophila* | HC79/132 | AF357055 | AF223212 |
| *Tephrocybe ambusta* | CBS452.87 | AF357057 | AF223216 |
| *Tephrocybe atrata* | CBS709.87 | AF357053 | AF223210 |
| *Tephrocybe gibberosa* | CBS328.50 | AF357041 | AF223197 |
| *Tephrocybe tylicolor* | BSI92/245 | AF357040 | AF223195 |
| *Tephrocybe palustris* | CBS717.87 | AF357044 | AF223200 |
| *Tricholomella constricta* | HC84/75 | AF357036 | AF223188 |

**Note:** The newly obtained sequences in this study are marked in bold.
